# Prevalence of sarcopenia in patients with COPD through different musculature measurements: An updated meta-analysis and meta-regression

**DOI:** 10.3389/fnut.2023.1137371

**Published:** 2023-02-16

**Authors:** Jie He, Hezhi Li, Jun Yao, Yan Wang

**Affiliations:** ^1^Clinical Medical College of Chengdu Medical College, Chengdu, Sichuan, China; ^2^Department of Pulmonary and Critical Care Medicine, The First Affiliated Hospital of Chengdu Medical College, Chengdu, Sichuan, China; ^3^Department of Anesthesiology, The First Affiliated Hospital of Chengdu Medical College, Chengdu, Sichuan, China

**Keywords:** sarcopenia, chronic obstructive pulmonary disease, meta-analysis, prevalence, lung function

## Abstract

**Aim:**

Chronic obstructive pulmonary disease (COPD) patients vary widely in terms of the prevalence of sarcopenia, which is partially attributed to differences in diagnostic criteria and disease severity. There are several different musculature measurements that are used to quantify sarcopenia. This study included published literature for meta-analysis to assess the sarcopenia prevalence in COPD patients and correlate the disease with the clinical characteristics of such patients.

**Methods:**

A comprehensive review of the English and Chinese literature on sarcopenia prevalence in COPD patients was conducted using electronic databases such as China National Knowledge Infrastructure (CNKI), Web of Science, Cochrane Library, EMBASE, PubMed, and Wanfang. Two researchers analyzed the studies for Newcastle-Ottawa Scale. The software Stata 11.0 was employed for the analysis of the acquired data. The standard mean differences method was utilized for the estimation and quantification of the effect size. Furthermore, a fixed- or random-effects model was employed for conducting a combined analysis.

**Results:**

In total, 56 studies were included as per the specific inclusion criteria. The resulting data of the assessed COPD patients in this research indicated a 27% prevalence of sarcopenia. Further analysis of subgroups was executed per disease severity, ethnicity, diagnostic criteria, gender, and age. Per these findings, increased disease severity elevated the prevalence of sarcopenia. The Latin American and Caucasian populations indicated an increased prevalence of sarcopenia. In addition, the prevalence of sarcopenia was related to diagnostic criteria and definition. Male COPD patients had a higher prevalence of sarcopenia than female COPD patients. COPD patients with an average age greater than 65 had a slightly higher prevalence of sarcopenia. COPD patients with comorbid sarcopenia had poorer pulmonary function, activity tolerance, and clinical symptoms than patients with COPD alone.

**Conclusion:**

Sarcopenia prevalence is high (27%) in COPD patients. In addition, these patients had worse pulmonary function and activity tolerance compared to patients without sarcopenia.

**Systematic review registration:**

https://www.crd.york.ac.uk/prospero/display_record.php?RecordID=367422, identifier CRD42022367422.

## 1. Introduction

Characterized by abnormal extra-pulmonary manifestations and chronic inflammation, chronic obstructive pulmonary disease (COPD), negatively affects the quality of life of patients and physical function (decreased physical activity endurance levels, strength, and mass of muscles) (1, 2). These aforementioned factors are also strongly associated with the onset and progression of sarcopenia. Sarcopenia is defined as a syndrome of progressive age-related decline in the strength and mass of muscles, with or without low physical performance. Notably, sarcopenia-related changes are not restricted to the muscle itself, as neurological and endocrine disorders are also linked to sarcopenia (3). The evaluation of sarcopenia mainly concerns to assess muscle mass, muscle strength, and physical performance, which was measured by dual-energy X-ray absorptiometry (DXA), bioelectrical impedance analysis, handgrip dynamometry, gait speed, etc. (3). Increased falls, hospitalization, and death are all highly correlated with sarcopenia, a primary cause of frailty in the elderly population (4), with a prevalence of approximately 5–10% in people older than 65 years of age (5). Moreover, Asian people appear to have greater sarcopenia prevalence than other regions (6).

Patients with COPD are at a significantly high risk of developing sarcopenia, with prevalence estimates ranging from 15 to 55% (7, 8). Sarcopenia prevalence of COPD is often associated with different musculature measurements, diagnostic criteria, ethnicity and disease severity, etc. According to a cross-sectional investigation of a Southeast Asian population, DXA was used to assess muscle mass, and 24% of individuals with COPD were found to have sarcopenia (6). Limpawattana et al. (6) pointed out that COPD patients often had relative or an absolute increase in fat mass which might lead to systemic inflammation, loss of fat-free mass, and insulin resistance which were significantly related to sarcopenia. Furthermore, increased systemic inflammation, and the possible presence of hypoxia and the more frequent use of systemic corticosteroids also resulted in excessive apoptosis of skeletal muscle (9). Thus, patients with COPD generally have a higher sarcopenia prevalence. In patients with concurrent COPD and sarcopenia, sarcopenia appears to negatively affect functional and health-related clinical indicators, and the increase in sarcopenia prevalence is linked to the severity of COPD (10). Sarcopenia can also lead to an increased risk of non-alcoholic liver disease, metabolic syndrome, and osteoporosis in COPD patients (11–13). Lower muscle strength also predicts an increased risk of acute exacerbations in COPD patients (14). Weakness of the diaphragm and the accessory muscle of inspiration is universally found in patients with long-duration COPD. Weakness of inspiratory muscle results in dyspnea and a decrease in exercise tolerance, as well as hypoxemia, in patients with COPD (15). All of these findings suggest that COPD and sarcopenia may have a mutually reinforcing relationship that leads to a vicious cycle. The actual clinical impact of sarcopenia in COPD remains to be elucidated despite its correlation with the worsening prognosis in COPD patients (16). However, it is challenging to adequately assess this impact due to the broad range of sarcopenia prevalence estimates in COPD.

Based on observational data, the most recent research has demonstrated wide-ranging and inconsistent results. Sepúlveda-Loyola et al. (17) conducted a meta-analysis involving literature published between 2006 and 2018 in 2020 analyzing sarcopenia prevalence in COPD patients and comparing the variances in pulmonary function and activity tolerance between patients with sarcopenia and those without sarcopenia. The main sites included in the previous meta-analyses were developed countries, such as Europe and North America. As China is also one of the countries with a high burden of COPD (18), therefore, the inclusion of studies involving patients from the Chinese population in the meta-analysis is necessary. Different musculature measurements used for the diagnosis of sarcopenia may lead to the different prevalences of sarcopenia in patients with COPD. In addition, several studies on concurrent COPD and sarcopenia have been conducted in recent years, yielding widely varying results. Therefore, this meta-analysis aimed to include the most recent observational studies to determine the differences in sarcopenia prevalence in the different subgroups of COPD patients.

## 2. Methodology

### 2.1. Data source and search strategy

**FIGURE 1:**
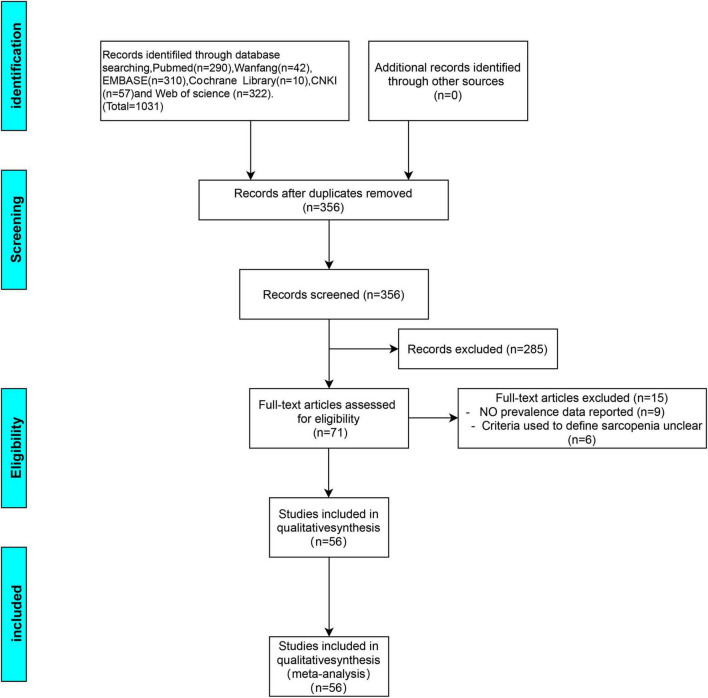
Preferred reporting item for meta-analysis (PRISMA) flow diagram for the study selection process.

This review’s protocol was registered in PROSPERO (CRD42022367422).^1^ From their establishment to October 2022, the following free-text and subject heading terms were utilized to search these six electronic databases [i.e., EMBASE, PubMed, Cochrane library, Web of Science, Wanfang, and China National Knowledge Infrastructure (CNKI)]: “COPD,” “chronic obstructive pulmonary disease,” “chronic obstructive lung disease,” “COAD,” “chronic obstructive airway disease,” and “sarcopenia.” To find more potential research, reference lists from included papers were manually searched. This research included papers based on whether they ultimately involved a sarcopenia diagnosis (defined per any criteria given that it was described in the methodology) and whether the COPD participants were adults regardless of the severity of the disease. Disease severity was assessed *via* the Global Initiative for Chronic Obstructive Lung Disease (GOLD) staging system (19). The nature of the study’s research question allowed for the inclusion of cross-sectional studies and clinical trials (whether randomized or not) and observational (e.g., cohort) studies. It was not possible to include an abstract or works published in languages other than English, Spanish, or Portuguese. The Preferred Reporting Items for Systematic Reviews and Meta-Analyses (PRISMA) flow diagram below (Figure 1) summarizes the results of the article selection processes.

This review’s key results were (i) the criteria utilized for defining sarcopenia and its prevalence, (ii) clinical indicators from studies that provided collected data between COPD patients who did and did not have sarcopenia. The clinical indicators were pulmonary function, modified Medical Research Council (mMRC) Dyspnea Scale score, BMI, obstruction, dyspnea, exercise capacity (BODE) index, and walking distance on the 6-min Walk Test (6MWT).

### 2.2. Data management and quality appraisal

The bibliographical reference manager software (EndNote 20, Thomson Reuters, Toronto, ON, Canada) was utilized to assemble the data resulting from examining the databases and the duplicates were eliminated. Two reviewers who worked independently analyzed the citations as per their abstract and title and determined their eligibility. They categorized these citations as either “include,” “exclude,” or “maybe” whose full text was further reviewed in order to reach a final yield, with an independent third reviewer resolving any conflict. This procedure was summarized per preferred reporting item for systematic reviews and meta-analysis recommendations. Using standardized templates suited to the objectives of the study, two team members extracted the data.

Using the Newcastle-Ottawa Scale (NOS) (20), which is commonly applied to examine case-control and cross-sectional studies, two researchers independently scored each study’s quality. NOS included study population selection (4 items), comparability (1 item), and exposure or outcome (3 items); scores for 0–3, 4–6, and 7–9 were thought to indicate low, medium, and high-quality studies, respectively. Higher scores indicate better methodological quality. Additionally, discussions between the two researchers helped in resolving their disputes.

### 2.3. Statistical analysis

The proportion of sarcopenia-associated COPD patients in each separate research (each research correlating to one estimate) was combined in a meta-analysis to determine the overall estimate of sarcopenia prevalence. An aggravated value, if it could be quantified, or the most “conventional” type was utilized in the case of the depiction of various sarcopenia types in a study (such as severe sarcopenia, sarcopenic obesity, and sarcopenia with normal body mass index). In the case of the use of more than one criterion for diagnosis (comparisons of different cut-off values with a single cohort) in a study, the estimates were summed up utilizing their stated main method or the one closely resembling the current the European Working Group on Sarcopenia in Older People (EWGSOP) recommendation (21) to avoid double counting. Subgroups were separately comparatively analyzed, where possible, through the χ^2^ test concerning the prevalence effect estimates between different elements such as disease severity (GOLD stages I–II vs. III–IV), gender (male vs. female), age, sarcopenia definitions (1 vs. >1 diagnostic criteria), and ethnicity (Caucasian, Latin American, and Asian). The Stata 11.0 (Stata Corporation, College Station, TX, USA) software *via* the function “metaprop” executed the meta-analysis with 95% confidence intervals (CIs) quantified through the score (Wilson) method as well as to account for the sarcopenia’s definitional variability across studies, a random-effects model (Dersimonian and Laird method).

Comparative analyses of COPD patients with and without sarcopenia were examined in terms of their resulting clinical outcomes, which were subjected to meta-analysis *via* Stata 11.0. Data in the form of weighted mean differences (WMD) and 95% CI were summarized. The primary technique of analysis was a random-effects model, with Deeks et al.’s interpretation of *I*^2^ statistic employed to characterize statistical heterogeneity (with values <25, 50–75, and >75% regarded as low, moderate, and high, respectively).

## 3. Results

The literature was searched systematically and thoroughly (Figure 1), and the databases provided 356 unique records, which were reduced to 56 articles (7, 8, 11, 16, 22–73) after the final review. Among these, 1 was a clinical non-randomized trial, 7 were observational cohort studies, and 48 had a cross-sectional design. In total, 28 reports provided comparative information on COPD patients with and without sarcopenia. The literature quality scores were summarized in Supplementary Table 1. The selected research’s characteristics were summarized in Table 1. This research covered various populations, such as Latinos, Asians, and Caucasians with 11, 24, and 21 studies from each, respectively.

**TABLE 1:** Characteristics of the included studies regarding the prevalence of sarcopenia in subjects with COPD.

References	Country	Study design	Sample size	Age (mean ± SD)	Male, *n* (%)	GOLD (%)	Prevalence of sarcopenia		Criteria (assessment method to detect sarcopenia)
							**Total**	**Male**	
Sergi et al. (50)	Italy	Cross-sectional	40	75.7 ± 5.3	40 (100)	–	0.38	1	LMM (DXA)
Gologanu et al. (51)	Romania	Cross-sectional	36	65.6 ± 7.5	12 (33)	I/II/III/IV (0/39/42/19)	0.08	–	LMM (BIA)
Koo et al. (52)	Korea	Cross-sectional	574	64.0 ± 0.6	574 (100)	I/II/III–IV (46/49/5)	0.27	1	LMM (DXA)
Chung et al. (11)	Korea	Cross-sectional	1,039	64.5 ± 9.4 (male), 64.5 ± 10.2 (female)	760 (73)	I/II/III/IV (46/48/5/1)	0.27	0.88	LMM (DXA)
Costa et al. (53)	Brazil	Cross-sectional	91	67.4 ± 8.7	41 (45)	I/II/III/IV (17/24/37/22)	0.4	0.56	LMM (DXA)
Jones et al. (8)	UK	Clinical non-randomized	622	–	354 (57)	–	0.14	0.63	LMM (BIA); LMS (HGS); LPP (4MGS)
van de Bool et al. (59)	Netherlands	Cross-sectional	45	65 ± 4	29 (64)	I/II/III/IV (6/36/49/9)	0.31	0.92	LMM (DXA)
Borda et al. (55)	Colombia	Cross-sectional	334		110 (30)	–	0.08	–	LMM (CC); LMS (HGS)
Cebron Lipovec et al. (7)	Netherlands	Prospective observational	112	66 ± 8	74 (66)	II/III/IV (17/52/31)	0.54	0.59	LMM (DXA); LPP (6MWT)
Joppa et al. (56)	12 countries and USA	Cross-sectional	2,000	63.5 ± 7.1	1,314 (66)	–	0.34	0.75	LMM (BIA)
Maddocks et al. (57)	UK	Prospective cohort	816	69.8 ± 9.7	484 (59)	–	0.12	–	LMM (BIA); LMS (HGS); LPP (4MGS)
Pothirat et al. (58)	Thailand	Cross-sectional	121	–	–	I/II/III/IV (26/25/10/39)	0.1	–	LMM (BIA)
van de Bool et al. (54)	Netherlands	Retrospective	505	65 ± 3	288 (57)	I/II/III/IV (13/41/40/11)	0.87	0.55	LMM (DXA)
Byun et al. (60)	Republic of Korea	Cross-sectional	80	68.4 ± 8.9	67 (83.8)	A/B/C/D (24/31/5/20)	0.25	0.85	LMS (HGS); LMM (BIA); LPP (6MWT)
Hwang et al. (61)	Korea	Cross-sectional	777	63.9 ± 10.6	777 (100)	I/II/III–IV (43/50/7)	0.053	1	LMM (DXA)
Kneppers et al. (62)	Netherlands	Prospective cohort	92	–	–	I/II/III/IV (3/25/50/23)	0.42	0.74	LMM (DXA)
Lee et al. (66)	Korea	Cross-sectional	748		–	–	0.34	0.74	LMM (DXA)
Limpawattana et al. (63)	Southeast Asia (Thailand)	Cross-sectional	121	70 ± 9	112 (92.6)	–	0.24	1	LMM (DXA); LMS (HGS); LPP (6MWT)
de Blasio et al. (64)	Italy	Cross-sectional	263	69.8 ± 8.0	185 (0.7)	I–II/III/IV (11/18/31)	0.24	–	LMM (BIA); LMS (HGS)
Munhoz da Rocha Lemos Costa et al. (65)	Brazil	Cross-sectional	121	67.9 ± 8.6	56 (46)	–	0.12	–	LMM (DXA)
Trajanoska et al. (22)	Netherlands	Cohort study	780	69.2 ± 9.1	–	–	0.09	–	LMM (DXA)
Lian (67)	China	Cross-sectional	96	62.48 ± 8.26	–	–	0.28	–	LMM (BIA)
Kovelis et al. (23)	Brazil	Cross-sectional	35	69.24 ± 1.54	20 (0.57)	II/III/IV (20/49/31)	0.2	–	LMM (DXA); LMS (HGS)
Machado et al. (24)	Brazil	Cross-sectional	270	–	–	–	0.2	–	LMM (BIA); LPP (6MWT)
Chua and Tee (25)	Philippines	Cross-sectional	24	69.38 ± 7.26	–	–	0.26	–	LMM (DXA); LMS (HGS); LPP (6MWT)
Demircioǧlu et al. (26)	Türkiye	Cross-sectional	219	66.9 ± 10.1	196 (89.5)	–	0.47	–	LPP (6MWT)
Perrot et al. (27)	France	Cross-sectional	54	68 ± 9		–	0.48	0.7	LMS (HGS)
Tsekoura et al. (28)	Greece	Cross-sectional	59	71.33 ± 7.48	10 (17)	–	0.29	–	LMM (BIA); LPP (4MGS)
Chi (68)	China	Cohort study	52	61.52 ± 10.23	26 (56)	–	0.37	0.31	LMS (HGS); LPP (6MWT)
Shi et al. (69)	China	Cohort study	220	68.03 ± 6.67	–	–	0.23	–	LPP (6MWT)
Attaway et al. (29)	USA	Cross-sectional	174,808	67.5 ± 14.5	5,929 (48.7)	–	0.07	–	LMM (BIA)
Espíndola de Araújo et al. (30)	Brazil	Prospective cohort	208	67.61 ± 10.05	94 (45.2)	I/II/III/IV (3/35/42/20)	0.16	0.2	LMM (BIA); LMS (HGS)
Hirai et al. (31)	Japan	Prospective observational	234	73.8 ± 7.9	234 (100)	–	0.2	1	LMM (BIA)
Kanezaki et al. (32)	Japan	Cross-sectional	60	76.9 ± 1.42	43 (72)	–	0.27	0.5	LMS (HGS); LPP (6MWT)
Lin et al. (33)	China	Cross-sectional	73	73.21 ± 9.54	59 (80)	–	0.38	0.71	LMM (DXA)
Sarwar et al. (34)	England	Cross-sectional	406	67.0 ± 4.5	186 (46)	–	0.07	–	LMS (HGS)
Schneider et al. (35)	Brazil	Cross-sectional	176	67 ± 8	96 (54.5)	I/II/III/IV (14/51/29/6)	0.21	0.7	LPP (6MWT)
Sepúlveda-Loyola et al. (36)	Brazil	Cohort study	39	69 ± 7	23 (59)	I–II/III–IV (46/54)	0.23	–	LMS (HGS); LMM (BIA)
van Beers et al. (37)	Netherlands	Cross-sectional	170	63.4 ± 9.4	91 (54)	0	0.3	0.47	LMS (HGS); LPP (6MWT)
Warnken-Miralles et al. (38)	Spain	Prospective	89	72 ± 10	70 (79)	–	0.25	0.7	LMS (HGS)
Hu et al. (72)	China	Cross-sectional	164	65.3 ± 5.5	93 (57)	–	0.32	–	LMS (HGS)
Ju et al. (73)	China	Cross-sectional	57	70 ± 7.2	50 (88)	–	0.18	–	LPP (6MWT)
Zhang et al. (71)	China	Cross-sectional	3,016	72 ± 8	1,512 (89)	–	0.27	0.27	LMM (DXA); LPP (6MWT); LMS (HGS)
Benz et al. (40)	Netherlands	Cross-sectional	681	69.1 ± 8.6	–	–	0.14	–	LMM (DXA)
Cao et al. (41)	Asian	Cross-sectional	20	–	–	–	0.45	–	LMM (BIA)
Deng et al. (42)	China	Cross-sectional	235	64.4 ± 10.7	160 (68)	I–II/III–IV (156/89)	0.35	0.33	LMM (BIA); LMS (HGS)
Erbas Sacar et al. (43)	Türkiye	Cross-sectional	29	–	–	–	0.14	–	LMS (HGS)
Gao et al. (44)	China	Cross-sectional	235	59.7 ± 16.6	83 (71)	I/II/III/IV (16/50/26/8)	0.36		LMM (BIA)
Kaluźniak-Szymanowska et al. (16)	Poland	Cross-sectional	124	69.4 ± 6.0	–	III–IV (52)	0.13	–	LPP (6MWT)
Lage et al. (39)	Brazil	Cross-sectional	43	73.9 ± 5.4	31 (72)	–	0.51	–	LMM (DXA)
Lage et al. (45)	Brazil	Cross-sectional	35	75.2 ± 3.2	24 (68.6)	I/II/III/IV (14/51/29/6)	0.57	0.7	LMM (DXA); LPP (6MWT)
Leem et al. (46)	Korea	Cross-sectional	704	62.4 ± 10.9	704 (100)	–	0.14	1	LMM (DXA)
Martínez-Luna et al. (47)	Mexico	Cross-sectional	185	72.20 ± 8.39	102 (55)	I–II/III–IV (57/43)	0.42	0.58	LPP (6MWT)
Sugiya et al. (48)	Japan	Cross-sectional	27	77.6 ± 4.6	23 (85)	I–II/III/IV (10/10/7)	0.22		LMS (HGS); LPP (6MWT)
Wang et al. (49)	Taiwan	Cross-sectional	44	51.2 ± 11.1	–	–	0.14	0.14	LMM (BIA); LMS (HGS)
Xu et al. (70)	China	Cross-sectional	220	72.9 ± 9.1	152 (69)	–	0.3		LMM (BIA); LMS (HGS)

GOLD, Global Initiative for Chronic Obstructive Lung Disease; 4MGS, 4 m gait speed; 6MWT, 6 min walking test; BIA, bioelectrical impedance analysis; CC, calf circumference; DXA, dual-energy X-ray absorptiometry; HGS, handgrip strength; LMM, lower muscle mass; LMS, lower muscle strength; LPP, lower physical performance; SD, standard deviation.

### 3.1. Assessment methods for sarcopenia

The selected research was assessed through the specific diagnostic criteria utilized for sarcopenia (Table 1). The diagnosis was based upon the measurement of low muscle mass (LMM) and low muscle strength (LMS) with the former solely used in 36 studies, and in 20 studies in combination with lower physical performance (LPP) and/or LMS. LMM, LMS, and LPP were identified through various methods and cut-off points in those studies. DXA, bioelectrical impedance analysis, and calf circumference were utilized to quantify muscle mass. Muscle strength was measured *via* handgrip dynamometry. Physical performance was measured *via* 6MWT. The various cut-off values utilized for defining each test’s “positive” results are depicted (Supplementary Table 2). Per the Asian group of sarcopenias (74) and EWGSOP (75) recommended threshold muscle mass, strength, as well as physical performance were mostly examined. The sarcopenia detection guidelines for COPD patients were comparatively analyzed (Supplementary Table 3).

### 3.2. Sarcopenia prevalence

**FIGURE 2:**
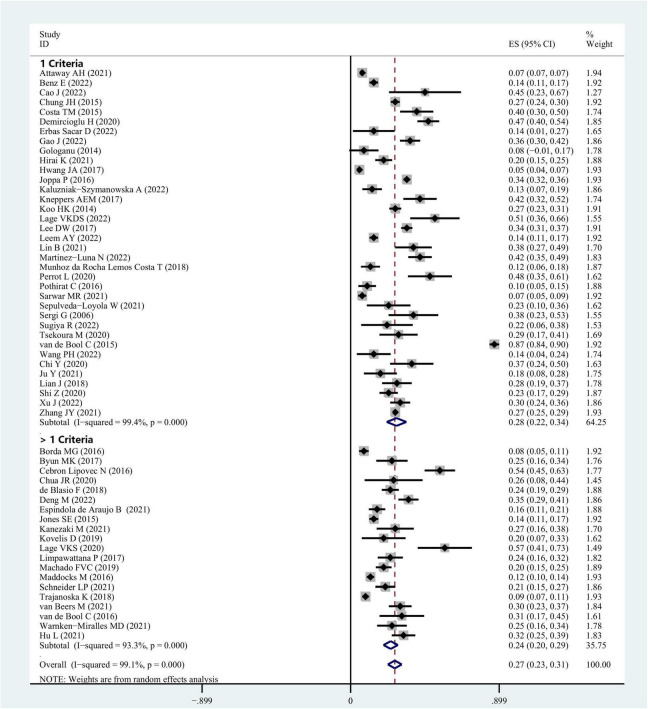
Prevalence of sarcopenia in COPD patients based on a random efforts model.

Concerning sarcopenia, the COPD patients’ overall pooled prevalence was estimated at 27% (95% CI = 0.23–0.31; Figure 2). This analysis depicted considerable statistical heterogeneity (*I*^2^ = 99.1%) implying that the individual study weighting was uniform (range 1.45–1.94%).

#### 3.2.1. Subgroup analysis

##### 3.2.1.1. Diagnostic criteria

Research that utilized only one criterion depicted considerably increased effect estimates [LMM; 28%, (95% CI = 0.22–0.34)] as compared to those that employed more than one criterion [LMM + LMS and/or LPP; 24% (95% CI = 0.20–0.29] (Figure 2 and Table 2).

**TABLE 2:** Subgroup analyses on the incidence of sarcopenia in various conditions.

Subgroup analysis (*n*)	ES (95% CI)	*P*-value	*I*^2^ (%)	P_*h*_
Overall (56)	0.27 (0.23–0.31)	<0.001	99.1	<0.001
**Ethnicity**				
Caucasian (21)	0.28 (0.19–0.77)	<0.001	99.5	<0.001
Asian (24)	0.25 (0.20–0.29)	<0.001	96.9	<0.001
Latino (11)	0.29 (0.20–0.37)	<0.001	93.8	<0.001
**Gender**				
Male (24)	0.35 (0.28–0.41)	<0.001	97.7	<0.001
Female (24)	0.28 (0.22–0.34)	<0.001	95.6	<0.001
**Criteria**				
1 criteria (36)	0.28 (0.22–0.34)	<0.001	99.4	<0.001
>1 criteria (20)	0.24 (0.20–0.29)	<0.001	93.3	<0.001
**GOLD**				
I–II (11)	0.28 (0.20–0.37)	<0.001	76.6	<0.001
III–IV (13)	0.39 (0.32–0.46)	<0.001	67.6	<0.001
**Age**				
Mean age<65 (10)	0.26 (0.18–0.36)	<0.001	98.6	<0.001
Mean age ≥65 (38)	0.28 (0.22–0.34)	<0.001	99.2	<0.001

P*_h_*, P*_heterogeneity_*; GOLD, Global Initiative for Chronic Obstructive Lung Disease.

##### 3.2.1.2. Gender

A higher sarcopenia prevalence was indicated in male [35% (95% CI = 0.28–0.41)] than in female [28% (95% CI = 0.22–0.34)] in studies concerning gender (Table 2).

##### 3.2.1.3. Disease severity

In some studies that provided data specific to disease severity, sarcopenia was found to be remarkably higher in patients with more severe disease [GOLD stages III–IV; 39% (95% CI = 0.32–0.46)] than those with less severe disease [GOLD stages I–II; 28% (95% CI = 0.20–0.37)] (Table 2).

##### 3.2.1.4. Ethnicity

Subgroup analysis was performed according to ethnicity. The combined prevalence values of concurrent COPD and sarcopenia were 28% (95% CI = 0.19–0.37), 25% (95% CI = 0.20–0.29), and 29% (95% CI = 0.20–0.37) among Caucasians, Asians, and Latinos, respectively (Table 2).

##### 3.2.1.5. Age

Next, we performed a subgroup analysis based on age. COPD patients with an average age greater than 65 had a slightly higher prevalence of sarcopenia [Mean age group ≥65 vs. Mean age <65 group: 28% (95% CI = 0.22–0.34) vs. 26% (95% CI = 0.18–0.36)] (Table 2).

#### 3.2.2. Meta-regression analysis and sensitivity analysis

**FIGURE 3:**
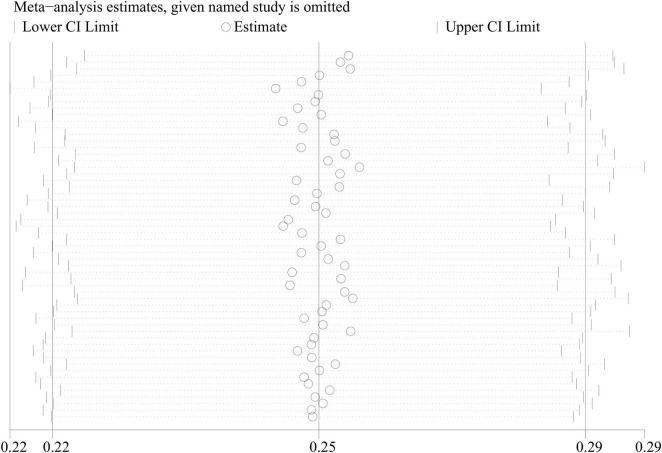
Sensitivity analysis of studies on the prevalence of sarcopenia in COPD patients in meta-analysis.

A combined analysis of the prevalence of sarcopenia in all COPD patients showed a high degree of heterogeneity (*I*^2^ = 99.1%, *P* < 0.001). Therefore, a meta-regression analysis was executed to determine the source of the high heterogeneity. Meta-regression analysis recorded *P*-values of 0.401, 0.176, 0.368, 0.442, and 0.598 for race, gender, disease severity, age, and diagnostic criteria, respectively, indicating no significant effect of these factors on heterogeneity. In the sensitivity analysis, each of the 56 studies was excluded in turn. Subsequently, a meta-analysis of the remaining studies was executed, and the results were compared with the results before the previous exclusion, indicating that the aforementioned exclusion of each study had no significant effect on the combined results (Figure 3).

### 3.3. Sarcopenia’s impact on clinical outcomes

#### 3.3.1. Pulmonary function

**FIGURE 4:**
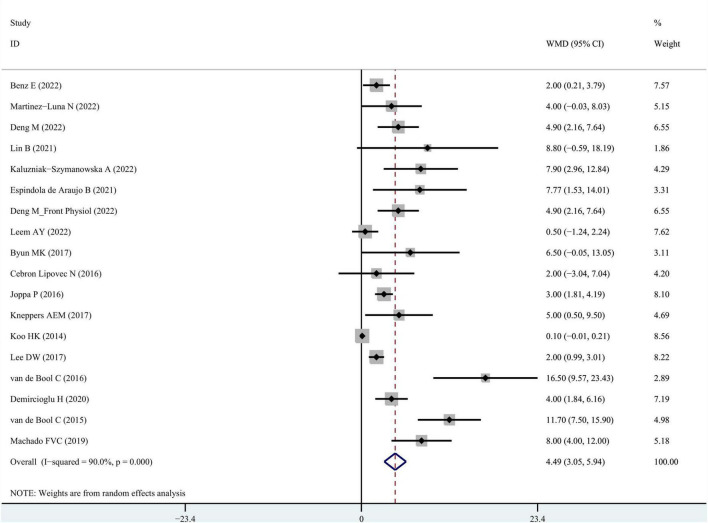
WMD forest plot and its 95% CI for FEV1/FVC in COPD group and sarcopenia + COPD group. FEV1, forced expiratory volume in 1 s; FVC, forced vital capacity; WMD, weighted mean differences.

A meta-analysis of pulmonary function data from 24 reports depicted that COPD patients with sarcopenia had poorer values on average concerning Forced Expiratory Volume in 1 s / Forced Vital Capacity (FEV1/FVC), predicted FEV1%, and predicted FVC% than those without sarcopenia (Table 3). The result of FEV1/FVC between the COPD group and the COPD + sarcopenia group was reported in Figure 4. The result of predicted FEV1% between the COPD group and the COPD + sarcopenia group was shown in Supplementary Figure 1. The result of predicted FVC% between the COPD group and the COPD + sarcopenia group was reported in Supplementary Figure 2.

**TABLE 3:** Meta-analysis, differences regarding lung function, 6MWT, mMRC, and BODE index between patients with and without sarcopenia.

Study (sarcopenia− vs. sarcopenia+)	WMD (95% CI)	*P*-value	*I*^2^ (%)	P_*h*_
FEV1/FVC (*n* = 18)	4.49 (3.05–5.94)	<0.001	90.0	<0.001
FEV1% predicted (*n* = 14)	9.14 (6.61–11.67)	<0.001	89.7	<0.001
FVC% predicted (*n* = 8)	6.11 (2.77–9.45)	<0.001	74.8	<0.001
6MWT (*n* = 11)	72.17 (45.11–99.23)	<0.001	83.3	<0.001
mMRC scores (*n* = 7)	0.47 (−0.81 to 0.13)	<0.001	87.6	<0.001
BODE index (*n* = 4)	−1.49 (−2.12 to −0.86)	<0.001	59.2	0.061

P*_h_*, P*_heterogeneity_*; FEV1, forced expiratory volume in 1 s; FVC, forced vital capacity; 6MWT, 6-min walk test; mMRC, modified Medical Research Council; BODE, body mass index, obstruction, dyspnea, and exercise capacity; WMD, weighted mean differences.

#### 3.3.2. Six-minutes walk test

**FIGURE 5:**
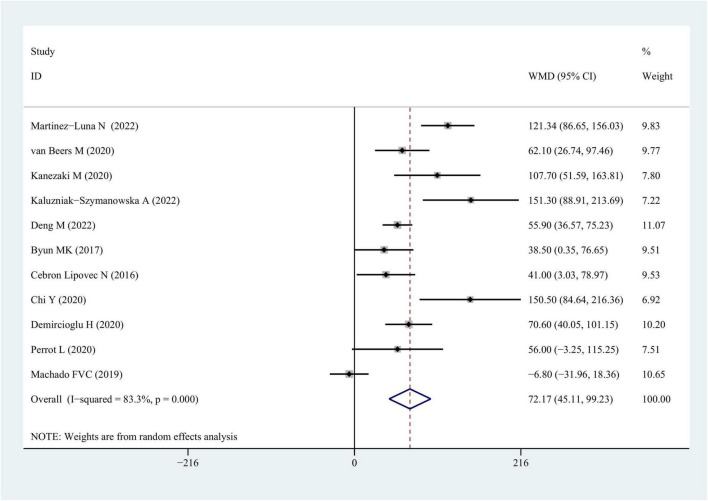
WMD forest plot and its 95% CI for the 6-min walk test in COPD group and sarcopenia + COPD group. WMD, weighted mean differences.

Data from 11 studies were available for outcomes related to 6MWT wherein a worse performance was linked to the sarcopenia-afflicted individuals in comparison with those lacking sarcopenia [COPD vs. COPD + sarcopenia: weighted mean difference = 72.17 (95% CI = 45.11–99.23), *P* < 0.001; *I*^2^ = 83.3%; Figure 5 and Table 3].

#### 3.3.3. mMRC scores

Seven studies involving reported data on mMRC scores were involved in the meta-analysis. The presence of sarcopenia was linked to mMRC scores [COPD vs. COPD + sarcopenia: weighted mean difference = −0.47 (95% CI = −0.81 to −0.13), *P* < 0.001; *I*^2^ = 87.6%; Supplementary Figure 3 and Table 3].

#### 3.3.4. BODE index

Four studies provided data on the BODE index in COPD patients without sarcopenia vs. those with sarcopenia, and the findings depicted that COPD patients had a lower BODE index than those in the sarcopenia group [COPD vs. COPD + sarcopenia: weighted mean difference: −1.49 (95% CI = −2.12 to −0.86), *P* < 0.001; *I*^2^ = 59.2%; Supplementary Figure 4 and Table 3].

## 4. Discussion

These systematic evaluations and meta-analyses provide unique insights into sarcopenia’s clinical relevance in patients with COPD. This study depicts the prevalence of sarcopenia in patients with COPD and the impact of gender, age, and different diagnostic criteria on its prevalence. Additionally, the present study compared the indicators reflecting pulmonary function and activity tolerance between the COPD group and the COPD + sarcopenia group.

Female COPD patients are slightly less likely to have comorbid sarcopenia than male COPD patients, which may be attributed to the higher proportion of men who smoke. Free radicals in cigarette smoke can lead to oxidative stress in skeletal muscle (76) and chronic smoke exposure can degenerate neuromuscular junctions (77). Regarding the different prevalences of sarcopenia between male and female in this meta-analysis, we believed smoking status might be one of the interfering factors. Unfortunately, we were not able to perform subgroup analysis by smoking status as included studies did not provide concrete data about sex differences in smoking.

In addition, the prevalence of concurrent COPD and sarcopenia varies between races and may be attributed to the large differences in race, body size, lifestyle habits, and muscle mass as well as strength measurements in different geographical populations. Moreover, we found that COPD patients with an average age greater than 65 had a slightly higher prevalence of sarcopenia. Sarcopenia is a common disease in the elderly population (78). Sarcopenia is a condition where muscle fiber cross-sectional area is reduced due to muscle atrophy and protein degradation (79). The occurrence of sarcopenia results from the combined action of internal and external factors. Age is considered an important contributor to sarcopenia, the skeletal muscle mass, myofiber size, and muscle strength endurance which reduce significantly with age (80). In addition, organismal inflammation increases during aging, which may excessively activate the ubiquitin-proteasome system (UPS). Protein degradation in skeletal muscle primarily depends on the activation of the two major proteolytic pathways, the UPS and the autophagy-lysosome system (81). Furthermore, aging perturbs organismal homeostasis and leads to multi-organ dysfunction and frailty, especially mitochondrial dysfunction, which has a central role in the occurrence of sarcopenia (82, 83). In this meta-analysis, the majority of included patients were older than 60 years, and several included studies did not provide information about the precise age. Thus, the difference in sarcopenia prevalence between the two groups (Mean age ≥65 group vs. Mean age <65 group) was not obvious. Therefore, more research into the non-elderly COPD group are required in the future.

Concerning sarcopenia diagnosis, two primary methods are currently used in patients with COPD: LMM-based independent assessment (50) and LMM-based assessment combined with LMS or LPP (21). The prevalence of sarcopenia in COPD patients diagnosed with LMM alone was 28%, while the value was 24% when diagnosed with LMM in combination with LMS and/or physiological function. Differences in prevalence due to different diagnostic criteria have been documented previously in older community-dwelling adults (84). Therefore, the different prevalences of concurrent COPD and sarcopenia reported in different studies may be partly due to the different diagnostic criteria for sarcopenia. This is not surprising, as increasing the number of restrictive conditions in the definition of sarcopenia will certainly decrease the incidence of a “positive” diagnosis. However, it may improve diagnostic accuracy. This is congruent with the current international diagnostic criteria, an important premise of which is the definition of sarcopenia as a geriatric syndrome indicated by LMM and LPP rather than by LMM alone. Only 20 of the included studies applied a sarcopenia definition that met these new criteria. According to the results of this study, increasing the severity of COPD elevated the probability of developing sarcopenia. Therefore, it seems sensible to stratify patients according to disease severity to guarantee as precise conclusions as possible from the current study. Combining the results of these studies may improve the understanding of the interactions between COPD and sarcopenia. International recommendations suggest DXA and bioelectrical impedance analysis as the preferred methods for assessing LMM at sites including the lower extremities and chest wall muscles (85). These testing methods are common in the literature included in this meta-analysis.

Regardless, normality or abnormality was assigned to test results per the observed various cut-off points. Advocated by the EWGSOP, the criteria of Baumgartner et al. (86) and Newman et al. (87) were the predominantly used criteria. Chen et al. (74) and Cruz-Jentoft et al. (75) did not support van de Bool et al. (54) measurement of muscle mass using calf circumference as a sarcopenia screening method, despite its simplicity. Such guidance is also present for measuring physical performance (gait speed) and muscular strength (handgrip force), although discrepancies were once again noticeable. A total of 4 m gait speed (51) and 6MWT (63) were employed for assessing the gait speed. Although both tests employed the same threshold for sarcopenia diagnosis (<0.8 m/s), they are both extremely different tests. A distance of 4 m is usually traversed at a normal walking speed in the 4 m gait speed (however, variances at various walk speeds and track lengths exist), while a walking track of 30 m is traversed in the 6MWT with the exercise tolerance of participants assessed by encouraging them to walk as far as they can [oftentimes faster than normal speed (88)]. There is a considerable risk that a measure of walking speed estimated from the 6MWT may be inaccurately interpreted. For instance, it was unable to differentiate between individuals moving slowly and those moving quickly but pausing to rest during the test. Therefore, it is possible that the prevalence of sarcopenia was underestimated in the research that adopted this strategy. Therefore, it is essential that future studies adopt standardized cut-off values to enable precise test interpretation in addition to using consistent diagnostic procedures to detect sarcopenia.

Sarcopenia has a persistent negative impact on a range of clinical features associated with COPD, such as balance, exercise capacity, grip strength, gait speed, and physical activity levels (53). It is also linked to an elevated symptom burden and a worse quality of life. It is noteworthy that the two studies that quantified dyspnea (Medical Research Council scale) (8, 60) classified sarcopenia solely per physical function, which is important since it suggests that functional impairment may be more strongly associated with dyspnea than LMM. Additionally, this brings up some difficult issues regarding the strategies employed for clinical management. Despite the correlation between COPD and sarcopenia, a causal relationship between the two diseases cannot be inferred. This research suggested that sarcopenia should be emphasized as an important “treatable trait” in adult respiratory medicine. Improvement in sarcopenia should serve as an important indicator of the efficacy of COPD rehabilitation. Jones et al. (8) indicated that an exercise-based, comprehensive intervention, pulmonary rehabilitation, was a multi-component technique that benefited a wide variety of clinical outcomes and decreased sarcopenia incidence in COPD patients. However, research is required to authenticate the findings of Jones et al. further, such as the use of nutritional supplementation and muscle strength training as recommended adjunctive therapies.

The actual mortality rate in patients with concurrent COPD and sarcopenia was not accurately quantified due to the lack of relevant literature. Nevertheless, it is speculated that sarcopenia may be associated with elevated mortality in patients with COPD due to the higher prevalence of sarcopenia in patients with GOLD stages III–IV. Leivseth et al. (89) reported that over 12 years of follow-up women depicted more than a sixfold increase in mortality risk while men depicted more than double the increase in risk in individuals with GOLD stages III and IV disease severity. Elevated fatality risk was indicated in COPD patients assessed *via* BODE, which is a widely used, valid tool for predicting the risk of death in COPD. In individuals with GOLD stage III and IV, Costa et al. (53) documented a rise in sarcopenia prevalence, with these quartiles linked to lower 4-year survival. A poorer physical and pulmonary function and quality of life were indicative of sarcopenia and these factors are known to be linked to an increased risk of death in patients with COPD. Multiple studies part of this meta-analysis reported data on pulmonary function, 6MWT, mMRC scale scores, and BODE scores comparing COPD patients with or without sarcopenia. The combined results revealed that COPD patients with comorbid sarcopenia had considerably lower projected FEV1 values than those without comorbid sarcopenia. In addition, the 6-min walk distance was notably lower in patients with comorbid sarcopenia than in patients without comorbid sarcopenia, and the mMRC score and BODE index was increased in patients with comorbid sarcopenia than in patients without comorbid sarcopenia. Considering that COPD is both an airway and systemic inflammatory disease, a high concentration of inflammatory factors could affect muscle atrophy and decreased muscle function (90, 91). A study involving 164 older adults suggested that interleukin-6 levels were negatively associated with skeletal muscle mass index and grip strength, whereas tumor necrosis factor-α and C-reactive protein levels were negatively associated with muscle mass and strength (92). In addition, mechanisms such as hypoxia and oxidative stress, myocyte mitochondrial dysfunction, and muscle metabolism disorders in COPD (93–96) can all produce pathological changes in skeletal muscle in terms of muscle volume and fiber type, ultimately leading to a significant decrease in muscle function. A definite relationship between sarcopenia and inflammatory biomarkers was not depicted because relevant data could not be extracted and obtained. Moreover, patients with acute exacerbations of COPD and severe COPD receive systemic applications of glucocorticoids that lead to negative nitrogen balance in the body and affect muscle mass and strength (97). Therefore, clinicians should be alert to the occurrence of sarcopenia and enhance screening for sarcopenia in patients with COPD to improve patients’ quality of survival and slow down the progression of the disease. In addition, patients with COPD should actively participate in endurance exercise training, pulmonary rehabilitation, and nutritional support to prevent the occurrence of sarcopenia.

This literature review has several limitations. First, factors such as different definitions of sarcopenia in different studies, subject characteristics, and diagnostic thresholds can lead to heterogeneity. Therefore, the interpretation of the results obtained from meta-analysis is challenging. Second, this review did not elucidate the direct relationship between sarcopenia and mortality due to the lack of data. Therefore, a long-term follow-up is needed to observe the survival time in the patient cohort. Nevertheless, the association observed between sarcopenia and mortality risk (assessed by the BODE index) is noteworthy. Third, this study lacks direct evidence highlighting the clinical impact of sarcopenia on healthcare costs, which is an important area to be addressed in future studies. Fourth, the different subtypes of sarcopenia (e.g., sarcopenic obesity and severe sarcopenia) may lead to some limitations in the clinical application of the results of this study. In addition, most of the included studies were cross-sectional studies. Consequently, a causal relationship between sarcopenia and COPD could not be determined.

## 5. Conclusion

Sarcopenia prevalence is high (27%) in COPD patients. The prevalence of sarcopenia differs in COPD patients according to musculature measurements, gender, disease severity, ethnicity, and age. In addition, the pulmonary function and physical performance of COPD patients with sarcopenia were significantly worse than that of COPD patients without sarcopenia. In further clinical work, attention should be paid to screening for sarcopenia in patients with COPD.

## Data availability statement

The original contributions presented in this study are included in this article/Supplementary material, further inquiries can be directed to the corresponding author.

## Author contributions

JH and HL developed and refined the research idea, performed the data collection and data analysis, prepared the first manuscript draft, and edited the manuscript. YW and JY validated the data collection, developed the research idea, and proofread the manuscript. All authors had made a substantial, direct, and intellectual contribution to the work and approved it for publication.

## Footnotes

1
https://www.crd.york.ac.uk/prospero/display_record.php?RecordID=367422

## Supplementary material

Supplementary Figure 1 WMD forest plot and its 95% CI for FEV1% predicted in the COPD group and sarcopenia + COPD group. WMD, weighted mean differences; FEV1, forced expiratory volume in 1 s.

Supplementary Figure 2 WMD forest plot and its 95% CI for FVC% predicted in the COPD group and sarcopenia + COPD group. WMD, weighted mean differences; FVC, forced vital capacity.

Supplementary Figure 3 WMD forest plot and its 95% CI for mMRC scores in the COPD group and sarcopenia + COPD group. WMD, weighted mean differences; mMRC, modified Medical Research Council.

Supplementary Figure 4 WMD forest plot and its 95% CI for BODE index in the COPD group and sarcopenia + COPD group. WMD, weighted mean differences.
