# Supplementary figures and images for: Prevalence of sarcopenia in patients with COPD through different musculature measurements: An updated meta-analysis and meta-regression
Source: Front Nutr. 2023 Feb 16;10:1137371. doi: 10.3389/fnut.2023.1137371 (PMC9978530; doi:10.3389/fnut.2023.1137371)

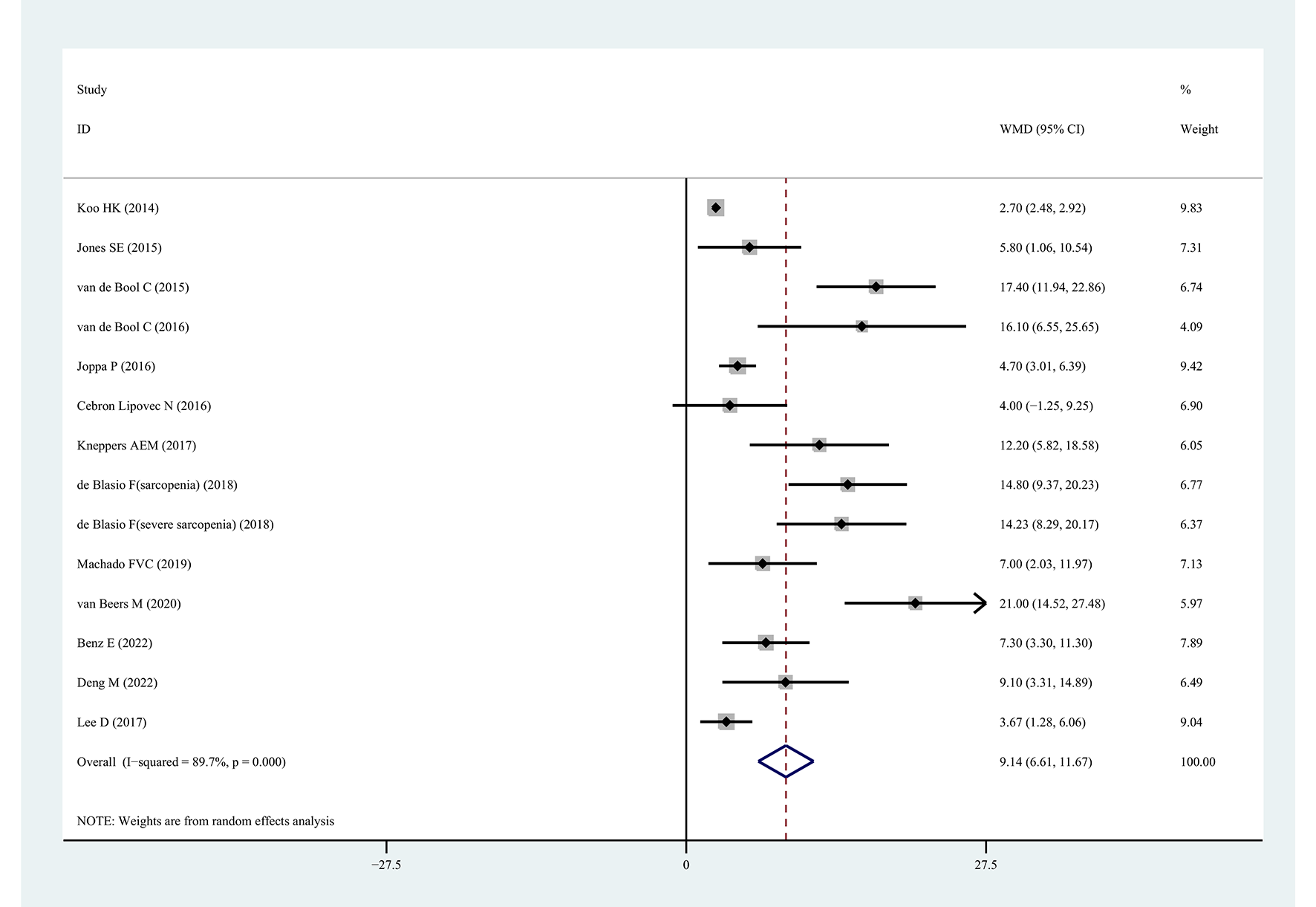

Supplement: Supplementary Figure 1 — WMD forest plot and its 95% CI for FEV1% predicted in the COPD group and sarcopenia + COPD group. WMD, weighted mean differences; FEV1, forced expiratory volume in 1 s. [file Image_1.TIF]

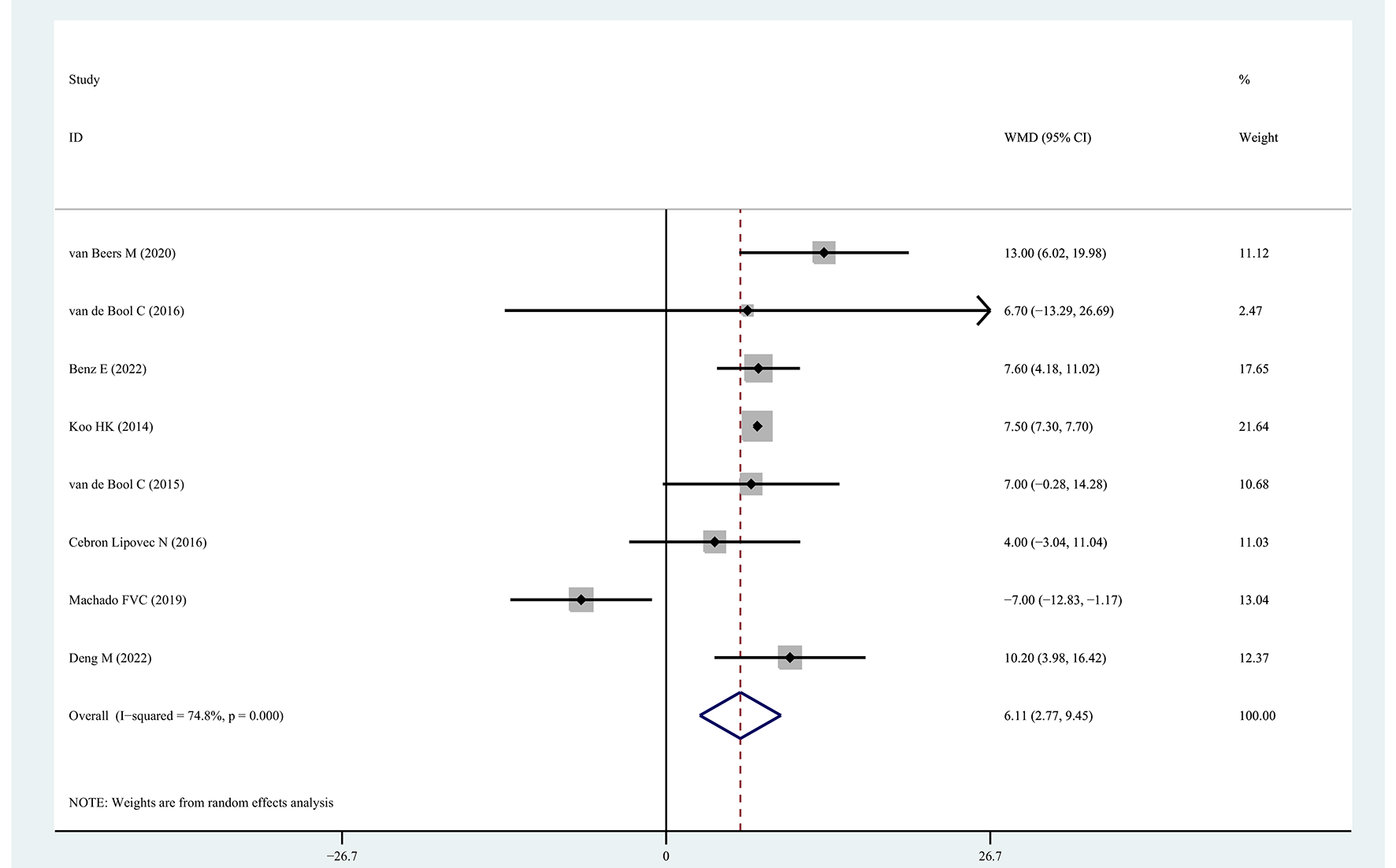

Supplement: Supplementary Figure 2 — WMD forest plot and its 95% CI for FVC% predicted in the COPD group and sarcopenia + COPD group. WMD, weighted mean differences; FVC, forced vital capacity. [file Image_2.TIF]

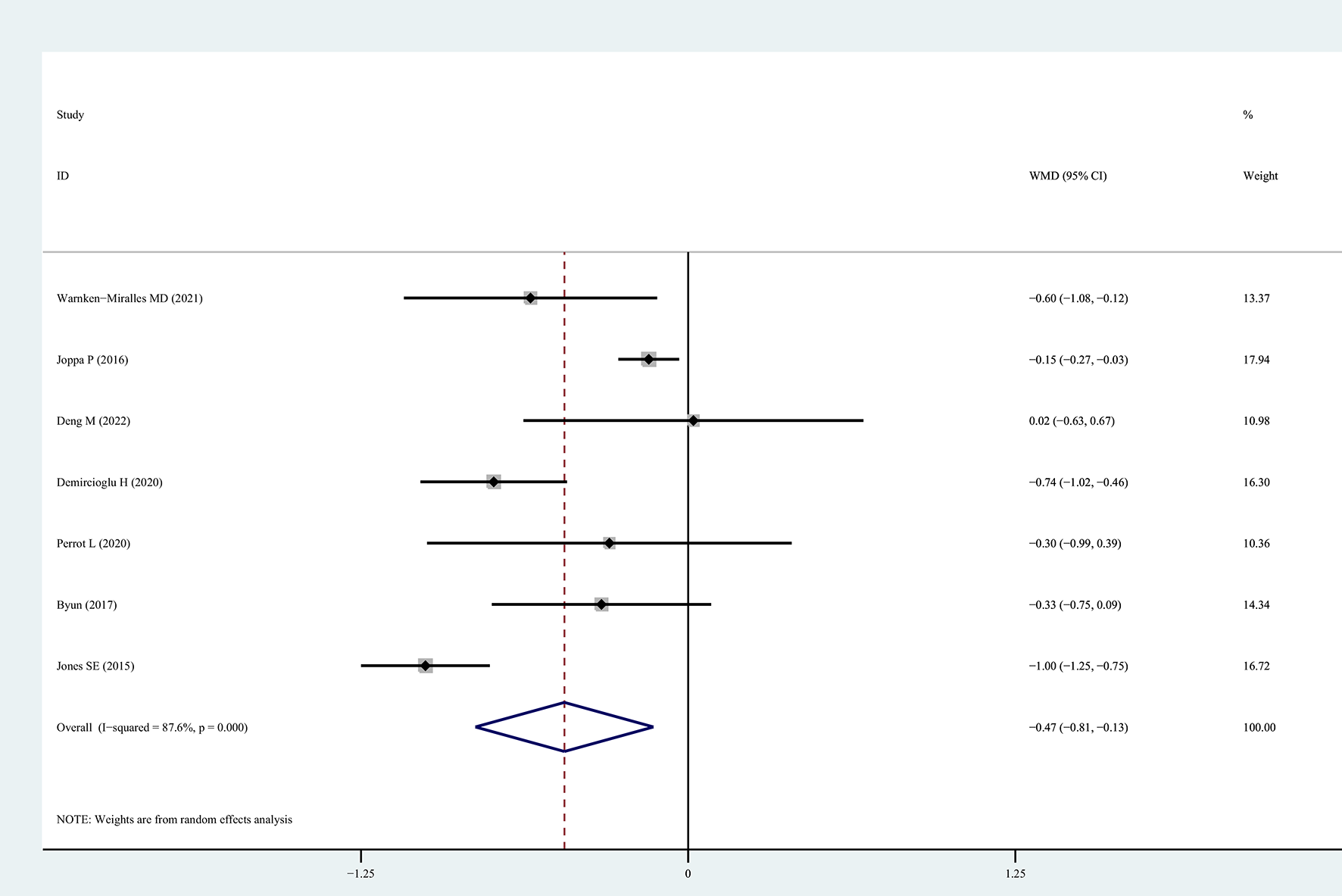

Supplement: Supplementary Figure 3 — WMD forest plot and its 95% CI for mMRC scores in the COPD group and sarcopenia + COPD group. WMD, weighted mean differences; mMRC, modified Medical Research Council. [file Image_3.TIF]

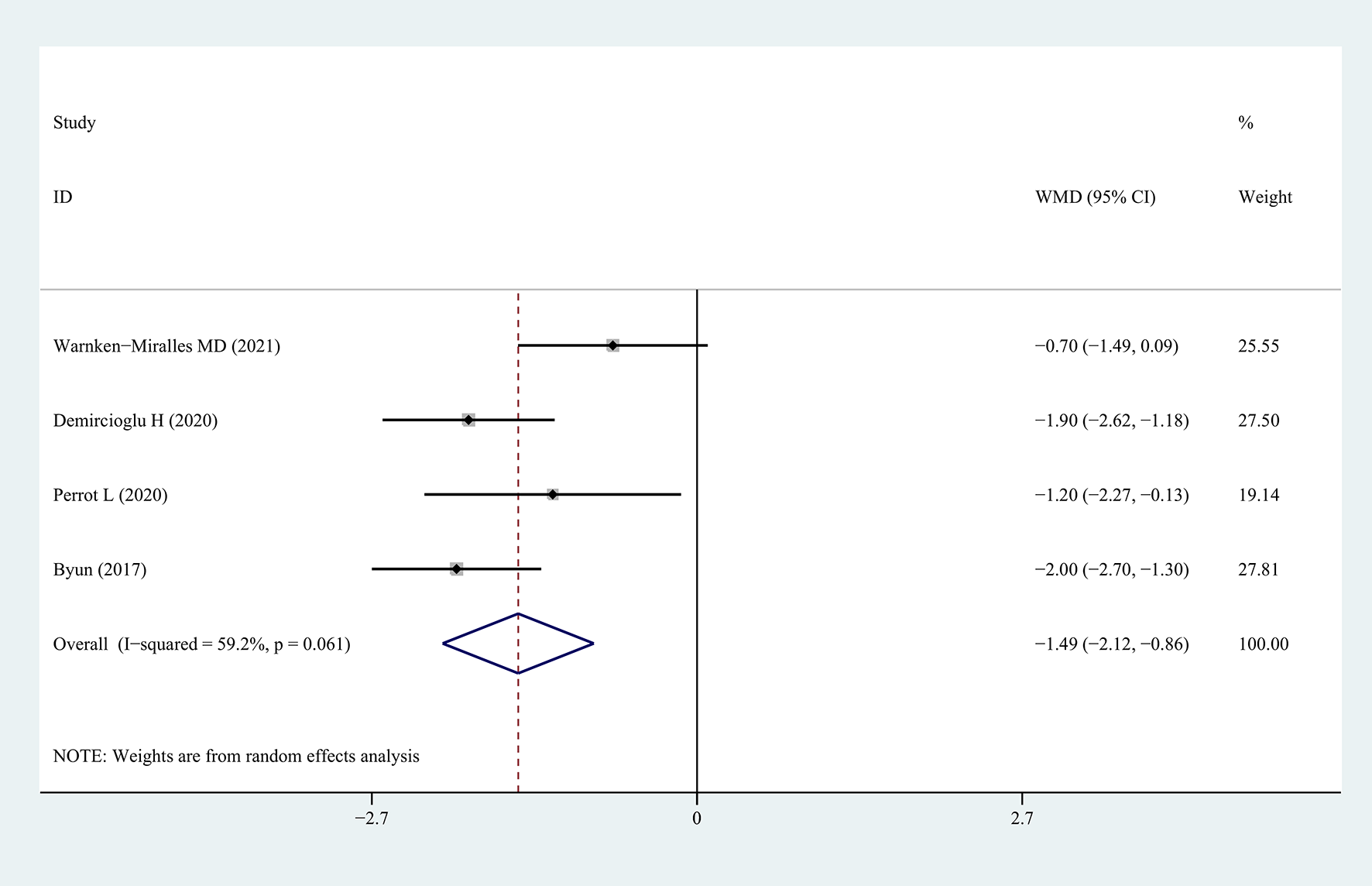

Supplement: Supplementary Figure 4 — WMD forest plot and its 95% CI for BODE index in the COPD group and sarcopenia + COPD group. WMD, weighted mean differences. [file Image_4.TIF]
