# Supplementary material for: Prevalence of sarcopenia in patients with COPD through different musculature measurements: An updated meta-analysis and meta-regression
Source: Front Nutr. 2023 Feb 16;10:1137371. doi: 10.3389/fnut.2023.1137371 (PMC9978530; doi:10.3389/fnut.2023.1137371)
Supplement: Supplementary file 5 [file Table_1.docx]

Supplementary Table 1: The literature quality scores of the included studies using the Newcastle-Ottawa Scale.

| First author and year | Selection | | | | Comparability | Outcome | | |
| --- | --- | --- | --- | --- | --- | --- | --- | --- |
|  | Q1 | Q2 | Q3 | Q4 | Q1 | Q1 | Q2 | Q3 |
| Trajanoska K（2018） | * | * | * | * | ** | ** | * |  |
| Kovelis D（2019） | * | * | * | * | ** | * | * |  |
| Machado FVC（2019） | * | * | * | * | ** | ** | * |  |
| Chua JR（2020） | * | * | * | * | ** | ** | * |  |
| Demircioğlu H（2020） | * | * | * | * | ** | ** | * |  |
| Perrot L (2020) | * | * | * | * | ** | ** | * |  |
| Tsekoura M (2020) | * | * | * | * | ** | ** | * |  |
| Attaway AH (2021) | * | * | * | * | ** | ** | * |  |
| Espíndola de Araújo B (2021) | * | * | * | * | ** | ** | * |  |
| Hirai K (2021) | * | * | * | * | ** | ** | * |  |
| Kaluźniak-Szymanowska A (2021) | * | * | * | * | ** | ** | * |  |
| Kanezaki M (2021) | * | * | * | * | ** | ** | * |  |
| Lin B (2021) | * | * | * | * | ** | ** | * |  |
| Sarwar MR (2021) | * | * | * | * | ** | ** | * |  |
| Schneider LP (2021) | * | * | * | * | ** | ** | * |  |
| Sepúlveda-Loyola W(2021） | * | * | * | * | ** | ** | * |  |
| van Beers M (2021) | * | * | * | * | ** | ** | * |  |
| Warnken-Miralles MD (2021) | * | * | * | * | ** | ** | * |  |
| Lage VKDS（2022） | * | * | * | * | ** | ** | * |  |
| Benz E（2022） | * | * | * | * | * | ** | * |  |
| Cao J（2022） | * | * | * | * | ** | ** | * |  |
| Deng M（2022） | * | * | * | * | ** | ** | * |  |
| Erbas Sacar D（2022） | * | * | * | * | * | * | * |  |
| Gao J（2022） | * | * | * | * | ** | ** | * |  |
| Lage VKS（2020） | * | * | * | * | ** | ** | * |  |
| Leem AY（2022） | * | * | * | * | ** | ** | * |  |
| Martínez-Luna N（2022） | * | * | * | * | ** | ** | * |  |
| Sugiya R（2022） | * | * | * | * | ** | ** | * |  |
| Wang PH（2022） | * | * | * | * | * | ** | * |  |
| Sergi G（2006） | * | * | * | * | ** | ** | * |  |
| Gologanu D（2014） | * | * | * | * | ** | ** | * |  |
| Koo HK（2014） | * | * | * | * | * | ** | * |  |
| Chung JH（2015） | * | * | * | * | ** | ** | * |  |
| Costa TM（2015） | * | * | * | * | ** | * | * |  |
| Jones SE（2015） | * | * | * | * | * | ** | * |  |
| van de Bool C（2015） | * | * | * | * | ** | ** | * |  |
| Borda MG（2016） | * | * | * | * | ** | * | * |  |
| Cebron Lipovec N（2016） | * | * | * | * | * | * | * |  |
| Joppa P（2016） | * | * | * | * | ** | ** | * |  |
| Maddocks M（2016） | * | * | * | * | ** | ** | * |  |
| Pothirat C（2016） | * | * | * | * | ** | ** | * |  |
| van de Bool C（2016） | * | * | * | * | ** | * | * |  |
| Byun MK（2017） | * | * | * | * | * | ** | * |  |
| Hwang JA（2017） | * | * | * | * | ** | * | * |  |
| Kneppers AEM（2017） | * | * | * | * | * | ** | * |  |
| Limpawattana P（2017） | * | * | * | * | ** | * | * |  |
| de Blasio F（2018） | * | * | * | * | ** | * | * |  |
| Munhoz da Rocha Lemos Costa T（2018） | * | * | * | * | ** | ** | * |  |
| Lee DW（2017） | * | * | * | * | ** | ** | * |  |
| Lian J (2018) | * | * | * | * | ** | ** | * |  |
| Chi Y (2020) | * | * | * | * | * | * | * |  |
| Shi M (2020) | * | * | * | * | * | * | * |  |
| Xu J (2022) | * | * |  | * | * | ** | * |  |
| Zhang JY (2021) | * | * | * | * | * | * | * |  |
| Hu L (2021) | * | * | * | * | * | ** | * |  |
| Ju Y (2021) | * | * |  | * | * | * | * |  |

NEWCASTLE - OTTAWA QUALITY ASSESSMENT SCALE

CASE CONTROL STUDIES

Note: A study can be awarded a maximum of one star for each numbered item within the Selection and Exposure categories. A maximum of two stars can be given for Comparability.

Selection

1) Is the case definition adequate?

a) yes, with independent validation 

b) yes, eg record linkage or based on self-reports

c) no description

2) Representativeness of the cases

a) consecutive or obviously representative series of cases 

b) potential for selection biases or not stated

3) Selection of Controls

a) community controls 

b) hospital controls

c) no description

4) Definition of Controls

a) no history of disease (endpoint) 

b) no description of source

Comparability

1) Comparability of cases and controls on the basis of the design or analysis

a) study controls for ______________ (Select the most important factor.) 

b) study controls for any additional factor. (This criteria could be modified to indicate specific control for a second important factor.)

Exposure

1) Ascertainment of exposure

a) secure record (eg surgical records) 

b) structured interview where blind to case/control status 

c) interview not blinded to case/control status

d) written self-report or medical record only

e) no description

2) Same method of ascertainment for cases and controls

a) yes 

b) no

3) Non-Response rate

a) same rate for both groups 

b) non respondents described

c) rate different and no designation
