# Supplementary material for: Prevalence of sarcopenia in patients with COPD through different musculature measurements: An updated meta-analysis and meta-regression
Source: Front Nutr. 2023 Feb 16;10:1137371. doi: 10.3389/fnut.2023.1137371 (PMC9978530; doi:10.3389/fnut.2023.1137371)
Supplement: Supplementary file 6 [file Table_2.docx]

| Supplementary Table 2 Different cut-off points used to identify Sarcopenia. | | | |
| --- | --- | --- | --- |
| Definition | **Lower MM** | **Lower MS** | **Lower PP** |
| EWGSOP  2010 | ASMI: <7.23 kg·m−2 for men and <5.67 kg·m−2 for women. | HGS: <30 Kg for men and < 20 Kg for women. |  |
| EWGSOP  2018 | ASM: ≤ 20 kg/m^2^ for men and ≤ 15 kg/ m^2^ for women. | HGS: <27 Kg for men and < 16 Kg for women. | GS ≤0.8 m/s |
| AWGS | ASMI: ≤ 7.0 Kg/m^2^ for men and ≤ 5.4 Kg/m^2^ for women. | HGS: <26 Kg for men and < 18 Kg for women. |  |
| FNIH | ALM/BMI: <0.789 for men and for < 0.512 women. | HGS: <26 Kg for men and < 16 Kg for women. |  |
| AWGS: Asian Working Group of Sarcopenia; EWGSOP: European Working Group of Sarcopenia in Older People; FNIH: The Foundation for the National Institutes of Health; MM: muscle mass; MS: muscle strength; PP: physical performance; HGS: handgrip strength; Kg: kilogram; GS: gait speed; ASMI: appendicular skeletal muscle index; ASM: appendicular skeletal muscle mass; ALM/BMI: appendicular lean mass/body mass index. | | | |
