# Supplementary material for: Prevalence of sarcopenia in patients with COPD through different musculature measurements: An updated meta-analysis and meta-regression
Source: Front Nutr. 2023 Feb 16;10:1137371. doi: 10.3389/fnut.2023.1137371 (PMC9978530; doi:10.3389/fnut.2023.1137371)
Supplement: Supplementary file 7 [file Table_3.docx]

Supplementary Table 3 criteria and cut-off points used to detect sarcopenia in individuals with COPD in the difficult studies

|  | Lower muscle mass | References |
| --- | --- | --- |
| DXA | 1. EWGSOP Newman et al 2003 ASMI: <7.23 kg/m^2^ for men and <5.67 kg/m^2^ for women. 2. EWGSOP Newman et al 2003 Residuals of linear regression on appendicular lean mass adjusted for fat as well as height. Men: -2.29, women: -1.73. 3. EWGSOP Baumgartner et al 1998 SMI: ≤7.26 kg/m^2^ for men and ≤5.45 kg/m2 for women. SMI: ≤7.26 kg/m^2^ for men 4. AWGS15 ASMI: ≤7.0 kg/m2 for men and ≤5.4 kg/m^2^ for women. 5. FNIH ALM/BMI: <0.789 for men and for < 0.512 women. 6. ASMMI: ≤ 2 standard deviations in a gender-specific mean for a young reference group. 7. SMI:<1 standard deviation in a gender-specific mean for a young reference group. 8. Combination of criteria 2 and 3. | Sergi G et al.2006、Koo HK et al.2014、Chung JH et al.2015、Costa TM et al.2015、van de Bool C et al.2015、Cebron Lipovec N et al.2016、van de Bool C et al.2016、Hwang JA et al.2017、Kneppers AEM et al.2017、Lee DW et al.2017、Limpawattana P et al.2017、Munhoz da Rocha Lemos、Costa T et al.2018、Trajanoska K et al.2018、Kovelis D et al.2019、Chua JR et al.2020、Lin B et al.2021  Zhang JY et al.2021 、Benz E et al.2022 、Lage VKDS et al.2022 、Lage VKS et al.2022、Leem AY et al.2022 |
| BIA | 1. EWGSOP7 Janssen et al 2002 SMI: ≤8.50 kg/m^2^ for men and ≤5.75 kg/m^2^ for women. 2. ATS BMI >21 and FFMI ≤16 kg/m^2^ for men or ≤15 kg/m^2^ for women. 3. Franssen et al 2014 Lower than the 10 percentiles of the reference value for age, sex, and BMI specific. 4. ASMMI: ≤2 standard deviations in a gender-specific mean for a young reference group. | Gologanu et al.2014、Joppa P et al.2016、Maddocks M et al.2016、Pothirat C et al.2016、Byun MK et al.2017、de Blasio F et al.2018、Lian J et al.2018、Machado FVC et al.2019、Tsekoura M et al.2020、Attaway AH et al.2021、Espíndola de Araújo B et al.2021、Hirai K et al.2021、Sepúlveda-Loyola W et al.2021、Cao J et al.2022、Deng M et al.2022、Gao J et al.2022、Wang PH et al.2022、Xu J et al.2022 |
| CC | Calf circumference <31 | Borda MG et al.2016 |
| Lower muscle strength | |  |
| HGS | 1. EWGSOP Laurentani et al 2003 HGS: <30 kg for men and <20 kg for women. 2. AWGS1 HGS: <26 kg for men and <18 kg for women. 3. Lower the last quintile in specific population. | Jones SE et al.2015、Borda MG et al.2016、Maddocks M et al.2016、Byun MK et al.2017、Limpawattana P et al.2017、de Blasio F et al.2018、Kovelis D et al.2019、Chua JR et al.2020、Perrot L et al.2020、Chi Y et al.2020、Espíndola de Araújo B et al.2021、Kanezaki M et al.2021、Sarwar MR et al.2021、Sepúlveda-Loyola W et al.2021、van Beers M et al.2021、Warnken-Miralles MD et al.2021、Hu L et al.2021、Deng M et al.2022、Erbas Sacar D et al.2022、Sugiya R et al.2022、Wang PH et al.2022、Xu J et al.2022 |
| Lower physical performance | |  |
| 4MGS | 1. EWGSOP7 Laurentani et al. 200347 GS: <0.8 m/s (both genders). | Jones SE et al.2015、Maddocks M et al.2016、Tsekoura M et al.2020 |
| 3.4MGS | 1. Lower the last quintile in specific population. | Borda et al.2016 |
| 6MWT | 1. AWGS Laurentani et al 2003 GS: <0.8 m/s (both genders). 2. EWGSOP Laurentani et al 2003 GS: <0.8 m/s (both genders). 3. FNIH GS: <0.8 m/s (both genders). | Cebron Lipovec N et al.2016、Byun MK et al.2017、Limpawattana P et al.2017、Machado FVC et al.2019、Chua JR et al.2020、Demircioğlu H et al.2020、Chi Y et al.2020、Shi Z et al.2020、Kanezaki M et al.2021、Schneider LP et al.2021、van Beers M et al.2021、Ju Y et al.2021、Zhang JY et al.2021、Kaluźniak-Szymanowska A et al.2022、Lage VKS et al.2022、Martínez-Luna N et al.2022、Sugiya R et al.2022 |

3.4 MGS, 3.4 m gait speed; 4MGS, 4 m gait speed; 6MWT, 6 min walking test; ASMI, appendicular skeletal muscle index; ATS, American Thoracic Society; AWGS, Asian Working Group for Sarcopenia; BIA, bioelectrical impedance analysis; BMI, body mass index; CC, calf circumference; DXA, dual-energy X-ray absorptiometry; EWGSOP, European Working Group on Sarcopenia in Older People; FNIH, The Foundation for the National Institutes of Health Sarcopenia Project; HGS, handgrip strength; SMI, skeletal muscle mass index
